# Different Inward and Outward Conduction Mechanisms in Na_V_Ms Suggested by Molecular Dynamics Simulations

**DOI:** 10.1371/journal.pcbi.1003746

**Published:** 2014-07-31

**Authors:** Song Ke, E. N. Timin, Anna Stary-Weinzinger

**Affiliations:** Department of Pharmacology and Toxicology, University of Vienna, Vienna, Austria; University of Illinois, United States of America

## Abstract

Rapid and selective ion transport is essential for the generation and regulation of electrical signaling pathways in living organisms. Here, we use molecular dynamics (MD) simulations with an applied membrane potential to investigate the ion flux of bacterial sodium channel Na_V_Ms. 5.9 µs simulations with 500 mM NaCl suggest different mechanisms for inward and outward flux. The predicted inward conductance rate of ∼27±3 pS, agrees with experiment. The estimated outward conductance rate is 15±3 pS, which is considerably lower. Comparing inward and outward flux, the mean ion dwell time in the selectivity filter (SF) is prolonged from 13.5±0.6 ns to 20.1±1.1 ns. Analysis of the Na^+^ distribution revealed distinct patterns for influx and efflux events. In 32.0±5.9% of the simulation time, the E53 side chains adopted a flipped conformation during outward conduction, whereas this conformational change was rarely observed (2.7±0.5%) during influx. Further, simulations with dihedral restraints revealed that influx is less affected by the E53 conformational flexibility. In contrast, during outward conduction, our simulations indicate that the flipped E53 conformation provides direct coordination for Na^+^. The free energy profile (potential of mean force calculations) indicates that this conformational change lowers the putative barriers between sites S_CEN_ and S_HFS_ during outward conduction. We hypothesize that during an action potential, the increased Na^+^ outward transition propensities at depolarizing potentials might increase the probability of E53 conformational changes in the SF. Subsequently, this might be a first step towards initiating slow inactivation.

## Author Summary

Voltage gated sodium channels are essential components of living cell membranes. They regulate the cell potential by facilitating permeation of ions across the membrane. In the past decades, studies revealed that the bacterial selectivity filter (SF) exhibits a constricted architecture lined with electronegative carboxyl oxygens of four glutamic acid side chains (EEEE motif), which repulse anions but attract Na^+^ ions. Crystal structures enable the investigation of structural dynamics with computational methods. Ion selectivity and conduction mechanisms between Na^+^, K^+^ and Ca^2+^ are progressively elucidated by molecular dynamics simulations and free energy calculations. The structural dynamics of the protein, especially the flexibility of SF and its fundamental role in kinetics underpinning ion selectivity, conduction and channel gating are less well understood. To shed light on this question, we use computational simulations to simulate ion conduction with membrane potentials. Our results suggest different dynamical behaviors of the EEEE locus and distinct ion distribution patterns in the SF with respect to permeating directionalities. These findings indicate a novel mechanism in differentiating reciprocal transitions of ion flow, preventing large sodium efflux during action potential initiation and may further suggest that increased flipping propensities at depolarizing potentials, might initially trigger channel slow inactivation.

## Introduction

Na^+^ flux through voltage gated sodium channels (Na_V_) is crucial for initiating action potentials in the membranes of electrically excitable cells. They mediate a variety of biological functions such as muscle contraction, propagation of nerve impulses, release of hormones and many more [1]. As a consequence, mutations in Na_V_ channels lead to a variety of channelopathies, such as congenital epilepsy, cardiac arrhythmias or chronic pain [2], [3].

Recently, homotetrameric crystal structures of several bacterial Na_V_ channels were successfully resolved [4]–[10], providing a tremendous opportunity to investigate the structure and function of these channels on the atomistic level. They are composed of four membrane spanning subunits and contain six transmembrane (TM) helices per subunit. Helices S1 to S4 form the voltage sensing module. Helices S5, P1 segments, the selectivity filter (SF) region, P2 segments and S6 helices, lining the inner pore cavity, form the pore module. The SF of most bacterial channels contains the amino acid sequence TLESW. The four glutamic acid side chains [11] form a high field strength binding site (HFS) [12] which is essential for ion selectivity. In eukaryotic sodium channels, this site consists of the amino acids motif DEKA.

The molecular mechanisms underlying ion conduction and selectivity in Na_V_ are beginning to emerge. Computational methods, particularly molecular dynamics (MD) simulations are extensively adopted to address these questions [13]–[23].

As reviewed recently [24], sodium ions were illustrated to spontaneously traverse the SF into the cavity with energy barriers between ∼2–5 kcal/mol. Compared to potassium coordination in K_V_ channels, sodium ions partially or fully preserve their first hydration shells [13], [15]–[17], [20], [22]. A loosely coupled knock-on mechanism with an average ion occupancy around two in the SF was predicted during ion conduction [14], [15], [21], [22]. The incoming ion repulses the present ion out of the SF. The wide radius (≥9 Å) of the SF enables double occupancy of ions at the same level [20], [21]. Further, Na^+^ vs. K^+^ [15], [16], [20], [20]–[22] and Na^+^ vs. Ca^2+^ [17], [20] discrimination studies were carried out. These studies revealed higher energy barriers in the SF for K^+^ and Ca^2+^ compared to Na^+^. Subsequently, non-equilibrium simulations were performed to investigate conduction under applied membrane potentials and to study kinetics [21], [22]. The estimated inward conductance rate successfully reproduced electrophysiology data [22].

A recent study by Chakrabarti et al. [23], suggested that conformational changes at the EEEE motif (corresponding to E177 in Na_V_Ab) might play an important role in ion conduction. However, this observation was not reported in other simulations, except for simulations using Ca^2+^ as a charge carrier [17]. In K^+^ channels, subtle structural changes in the SF, involving rotations around a highly conserved glycine residue result in different non-conductive conformations [25], [26]. This regulation of ion flow by conformational changes of the selectivity filter is termed C-type inactivation.

It is not clear to which extend structural changes at the EEEE locus in Na_v_ channels are crucial for conductance and inactivation in Na_v_ channels.

To investigate these issues, we conducted MD simulations using the open conformation of the bacterial sodium channel homologue Na_V_Ms (Magnetococcus sp. (strain MC-1)) [7] pore domain focusing on the structural changes of the SF during inward and outward conduction.

## Results

### Single channel conductance

The four-fold symmetrical structure of Na_V_Ms (pdb identifier: 4F4L) was generated using chain A (splayed outward by 25° rotation about its Ψ-bond at position T84), which creates an open pore with a diameter of ∼14 Å [7]. As described by Ulmschneider et al. [22], a harmonic restraint was exerted on the S5 and S6 TM helices to keep the gate in the open conformation throughout simulations. This structure was then embedded into a POPC lipid patch and duplicated in the Z direction (pore axis). A constant charge imbalance of four elementary charges (4 *e*) across each lipid bilayer between the central electrolyte bath and the two outer ones was maintained during simulation (supplementary movie S1) [27].

**Figure 1:**
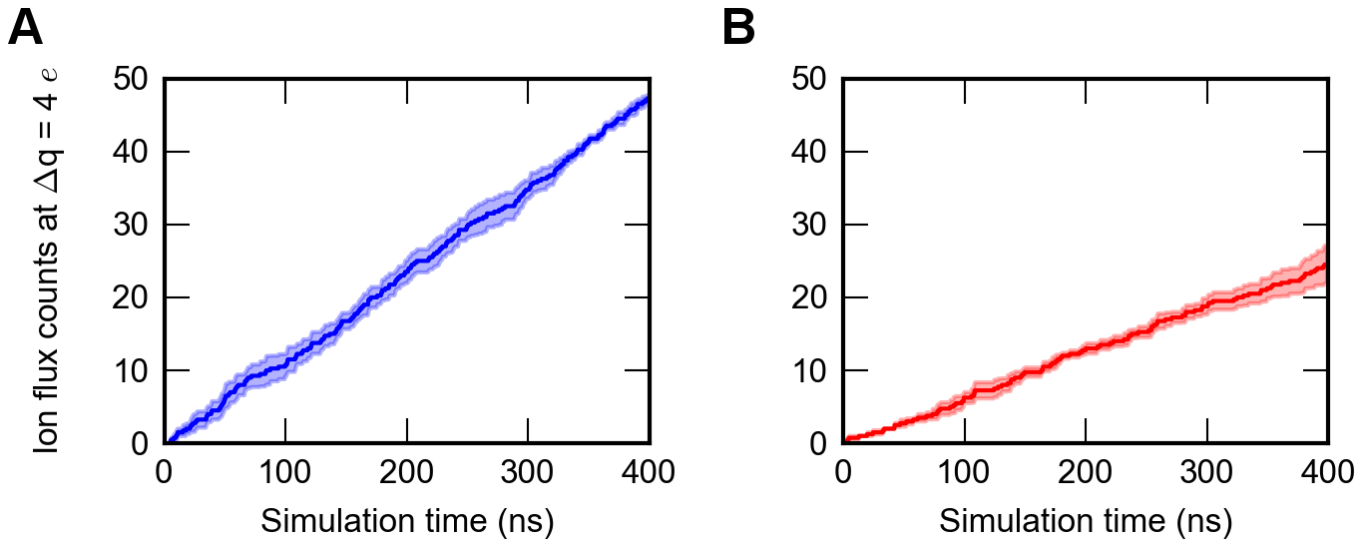
Ion flux in double bilayer simulations without dihedral restraints on E53 (n = 4). A) Average ion flux count of inward conduction through the SF over time (color: blue). B) Average ion flux count of outward conduction through the SF over time (color: red). Error estimations shown in the figure are S.E.M.

**Figure 2:**
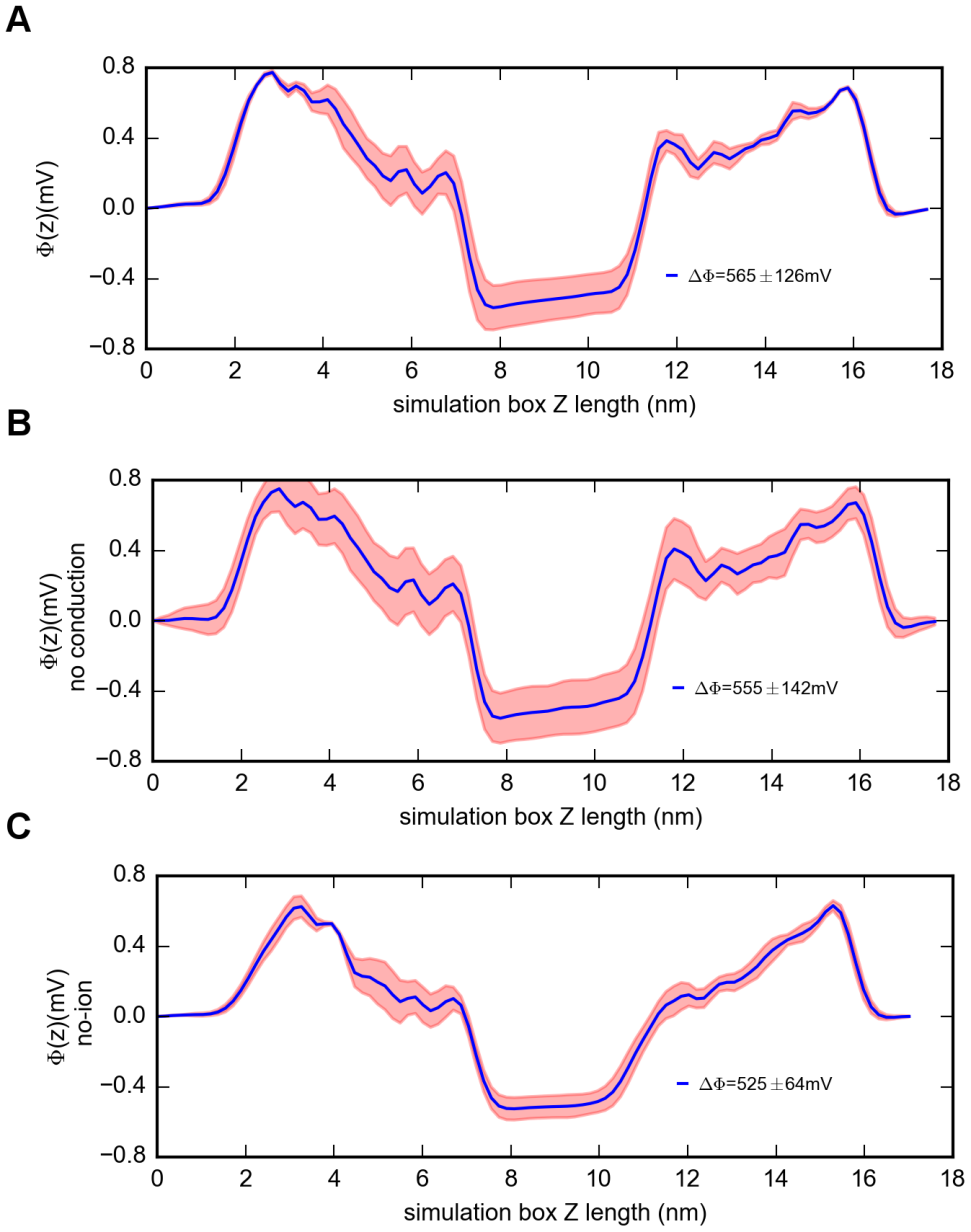
Transmembrane voltages comparison. A) Electrostatic potential across the simulation box calculated from the simulations without dihedral restraints on E53. B) Electrostatic potential across the simulation box calculated from the snapshots (t>5 ns) extracted from the simulation in Figure 2A without ion conduction events in neither inward nor outward directions. C) Electrostatic potential across the simulation box calculated from the “no salt” simulations. (Error estimations shown in the figure are S.D.).

Four times 500 ns double-patch MD simulations with 500 mM NaCl were performed, with the first 100 ns treated as equilibration. Figure 1 shows the cumulative ion conducting events from MD simulations with depolarized and hyperpolarized membrane potentials of ΔV: 565±126 mV (Figure 2A). The estimated sodium current in the inward direction is γ  =  27±3 pS. This value agrees with previously observed single channel conductance measurements (γ∼33 pS) [22]. Our double bilayer simulations enabled us to further estimate outward conduction, which amounts to γ  =  15±3 pS. Interestingly, this process is distinguishably slower than inward ion flux (P<0.01, N = 4).

### Ion distribution patterns and kinetics

**Figure 3:**
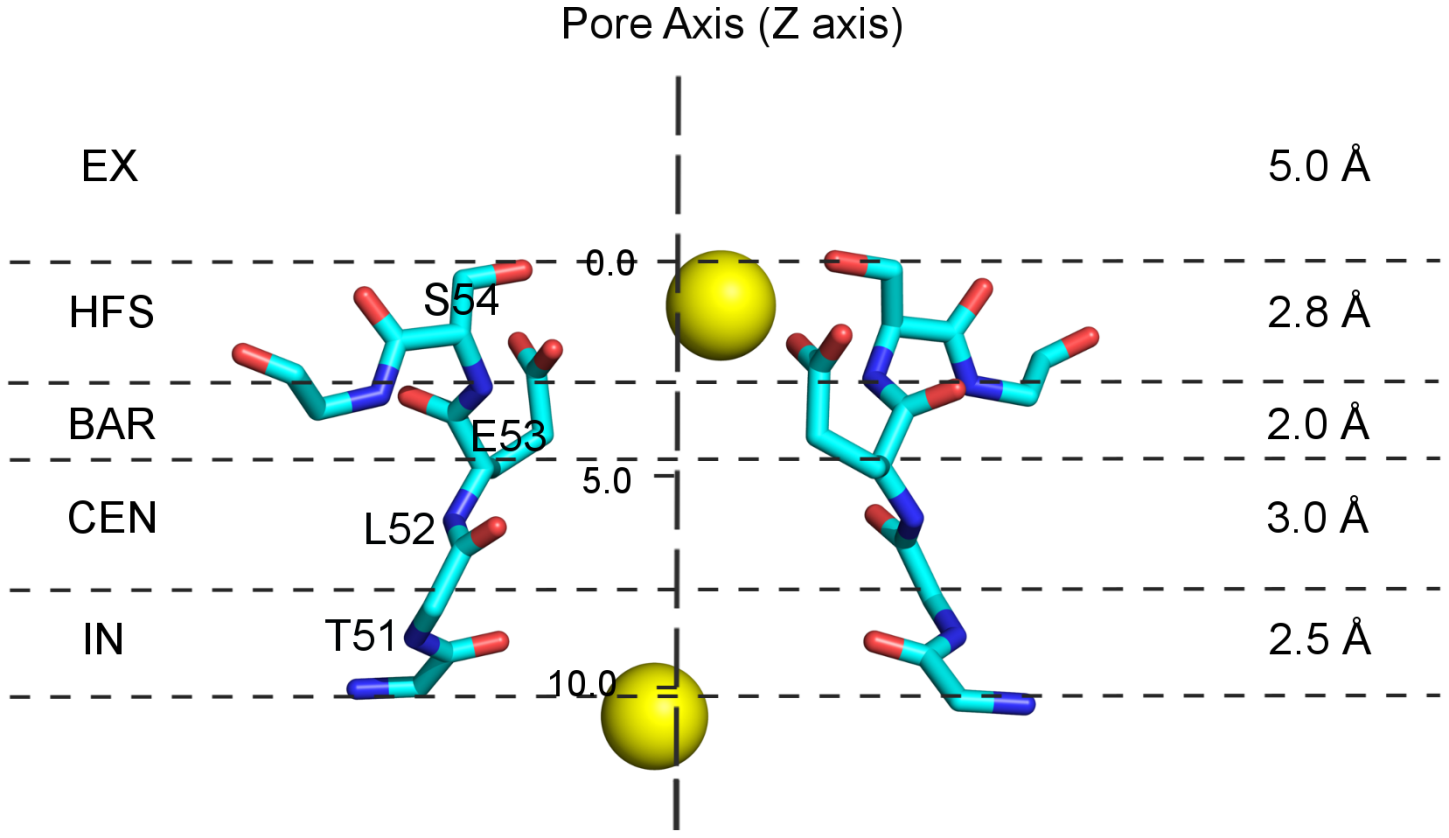
Annotations of the SF ion interacting site. SF backbone atoms are shown as blue sticks, E53 and S54 side chain atoms are also shown to determine the site S_HFS_ (only two opposite subunits are shown for clarity). In addition, sodium ions are depicted with yellow spheres. Sites S_EX_ (−5.00≤Z<0.00 Å), S_HFS_ (0.00≤Z<2.75 Å), S_BAR_ (2.75≤Z<4.75 Å), site S_CEN_ (4.75≤Z<7.75 Å) and site S_IN_ (7.75≤Z<10.25 Å) and the length of each site were annotated at the lateral sides of the figure.

To explore the underlying differences between inward and outward ion permeation, we plotted the ion probability density map across the pore region from all four simulations. Several favorable ion-interacting sites (S_EX_, S_HFS_, S_CEN_ and S_IN_) from periplasm to cytoplasm were assigned as proposed previously by Payandeh et al. [4]. The ion-interacting sites across the SF were determined by measuring the axial distance (Z axis) along certain atoms from −5.00 to 10.25 Å as shown in Figure 3. Side chain oxygens of S54 from all four chains were taken as the origin (Z = 0.0 Å) of the SF. Additionally, in two previous studies, a site with an energy barrier (∼2 kcal/mol) distinguishing between site S_HFS_ and S_CEN_ was identified [17], [22]. In this study, we refer to this site as “S_BAR_”, indicating this barrier (2.75≤Z<4.75 Å).

**Figure 4:**
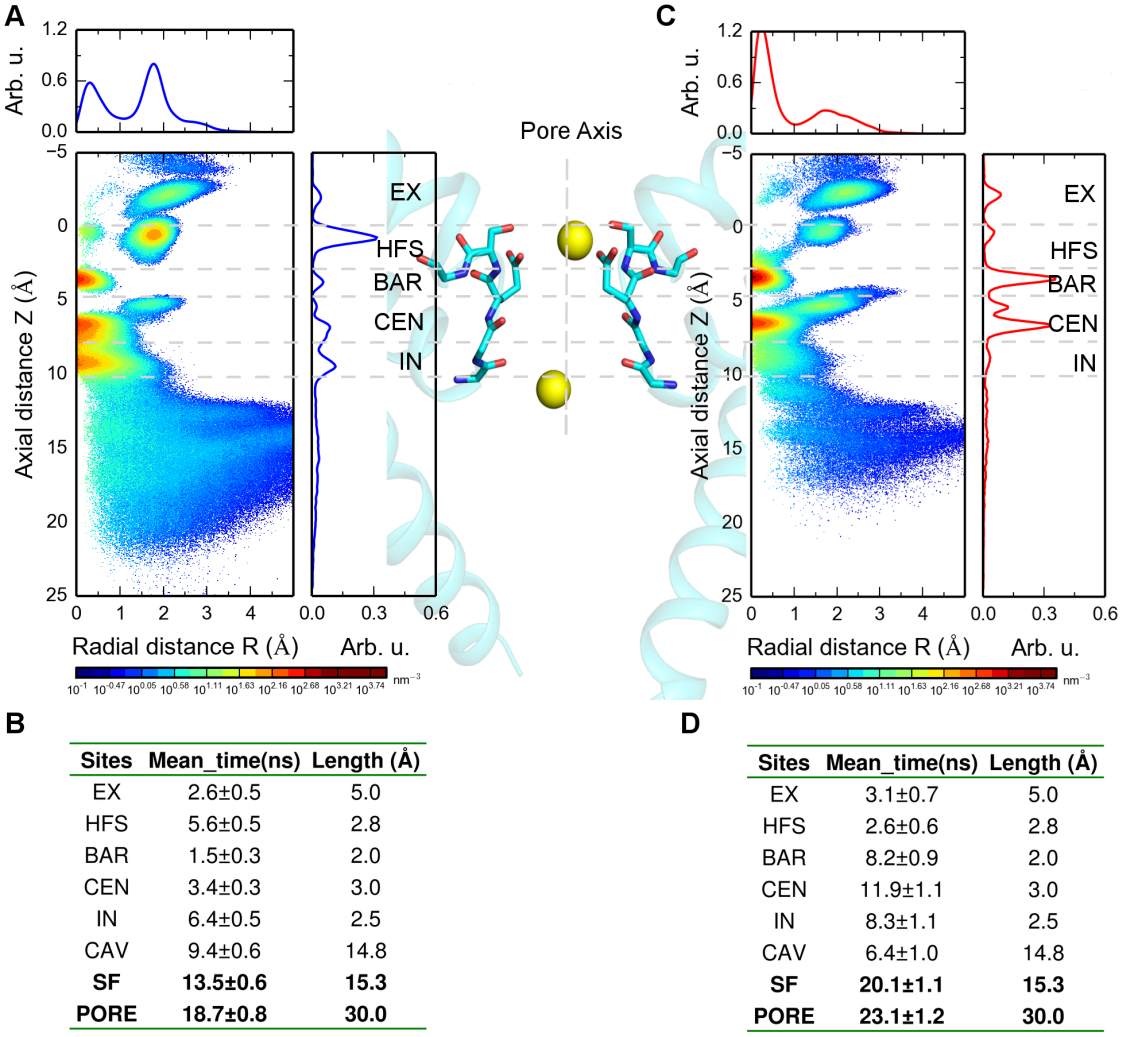
Ion distribution probability density map. A) Ion distribution of inward simulations labeled with respective interacting sites [4], lower left: 2-D (axial and radial distribution) density; lower right: 1-D axial distribution along the pore axis; upper left: 1-D radial distribution from the center of the pore axis. B) Inward dwell time in respective interacting sites (158 ion conduction events from four simulations were taken for analysis). C) Ion distribution of outward simulations labeled with respective interacting sites [4], lower left: 2-D (axial and radial distribution) density; lower right: 1-D axial distribution along the pore axis; upper left: 1-D radial distribution from the center of pore axis. D) Outward dwell time in respective interacting sites (79 ion conduction events from four simulations were taken for analysis).

During influx (Figure 4A and B), short-lived Na^+^ binding at site S_EX_ was observed (2.6±0.5 ns) in an asymmetrical manner. S_HFS_ is the dominant site with the highest ion density. At this site, ions tended to be directly coordinated with side chain oxygens of E53 and S54 in an off-axis manner. Additionally, a less densely populated configuration was observed in the center of this site consistent with previous studies [21], [24]. Moving inward from site S_HFS_, ions further translocated transiently via site S_BAR_ (1.5±0.3 ns) to site S_CEN_ (3.4±0.3 ns). Subsequently, ions reciprocally traversed between sites S_CEN_ and S_IN_. These results are in good agreement with previous simulation studies [14], [15], [17], [21], [22].

Our simulations revealed a distinct ion distribution pattern for efflux compared to influx as shown in Figure 4C and D. After entering into site S_IN_ from the cytosol, ions mainly populated sites S_CEN_ and S_BAR_ with extended dwell times compared to inward conduction, (S_CEN_: 11.9±1.1 ns vs. 3.4±0.3 ns; S_BAR_: 8.2±0.9 ns vs. 1.5±0.3 ns) suggesting a putative barrier for efflux between sites S_BAR_ and S_HFS_. Additionally, during efflux, Na^+^ ions tended to traverse in an on-axis manner through the filter.

### Structural dynamics of glutamic acid

**Figure 5:**
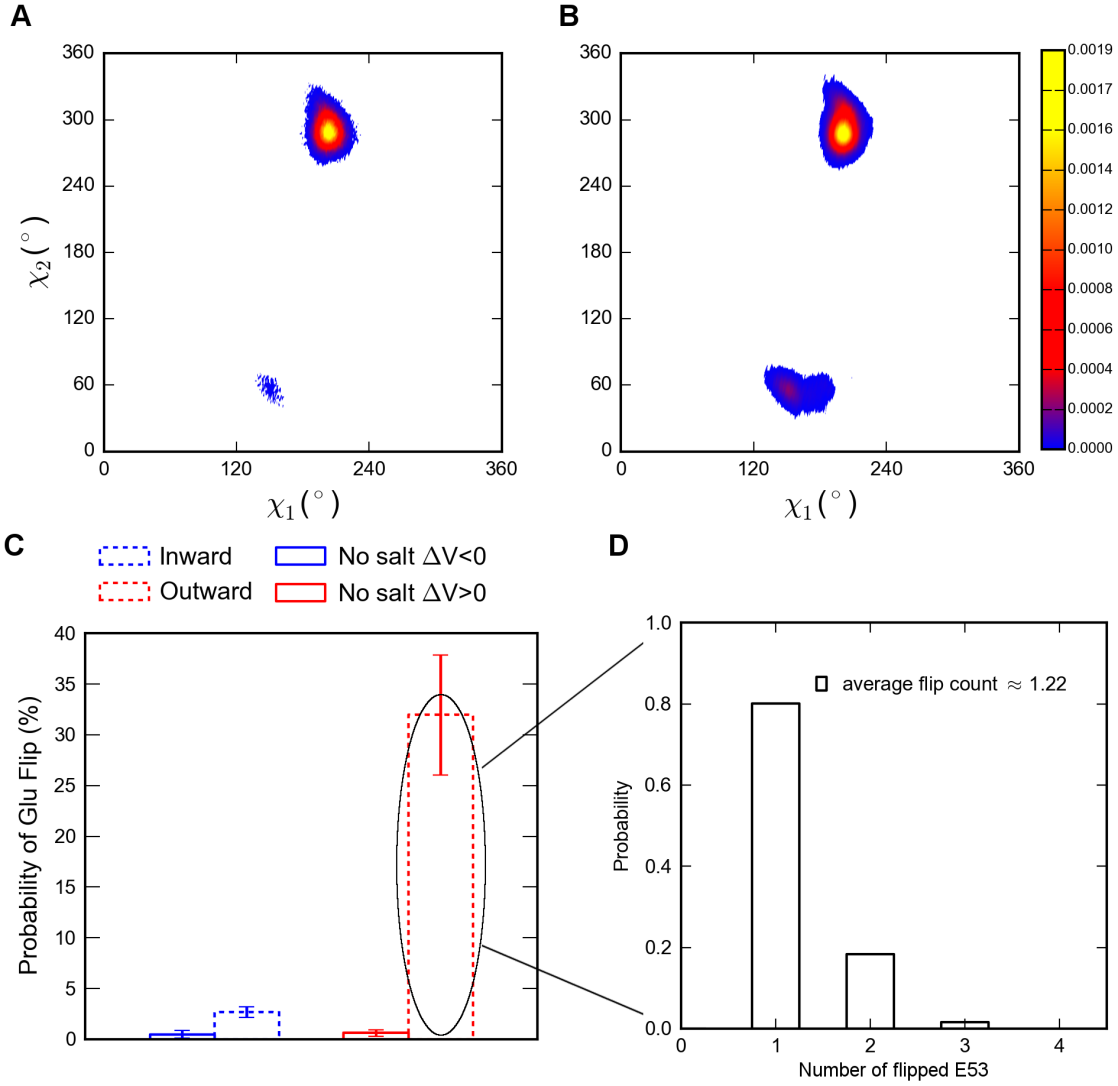
E53 conformational changes. A) χ_1_-χ_2_ angle distribution of the inward conductance simulations. B) χ_1_-χ_2_ angle distribution of the outward conductance simulations. C) The probability of flipping events of E53 side chains, the inward conduction is depicted as blue dotted histogram. The outward conduction is depicted as red dotted histogram (a flip is defined when at least one out of four subunits was shifted to flipping state in an analyzing window over time from four simulations); Control simulations with ions not in the SF (“no salt”) at hyperpolarized and depolarized membrane potentials are depicted as blue and red solid histograms, respectively. Error estimations shown in the figure are S.E.M. D) The distribution of E53 flipping numbers from all flipping events during outward conduction.

Conformational isomerization of the E53 side chains has been reported previously [17], [23]. Generally, the glutamic acid side chain might adopt two main conformations (Figure 5A and B): inward-facing (χ_2_ angle ∼60°, flipped) and outward-facing (χ_2_ angle ∼290°, non-flipped). In our simulations, during influx, flipping events were observed only in 2.7±0.5% of the simulation time, thus the E53 side chain mainly adopted a non-flipped conformation. In contrast, during efflux 32.0±5.9% flipping events were observed (P value  =  0.015, see Figure 5C). A more detailed investigation of this flipping events revealed that 80% of these changes occurred in only one of the four glutamic acid side chains (“one-flip”) (Figure 5D).

To investigate the influence of the presence of Na^+^ ions on E53 side chain dynamics, three repeated simulations without ions in the SF (“no salt”) were performed. Irrespective of the directionality of the applied potentials, the flip probability is less than 0.6% in all simulations (Figure 5C). This indicates, that a depolarizing potential per se does not significantly influence the number of E53 flipping events. This suggests that the combination of local positive charge carried by outward Na^+^ flux in the SF especially at sites S_HFS_ and S_BAR_ and the outward attracting membrane potential might collectively induce the rotation of the χ_2_ angle from ∼290° to ∼60°.

### Ionic binding modes and conduction mechanism during inward conduction

**Figure 6:**
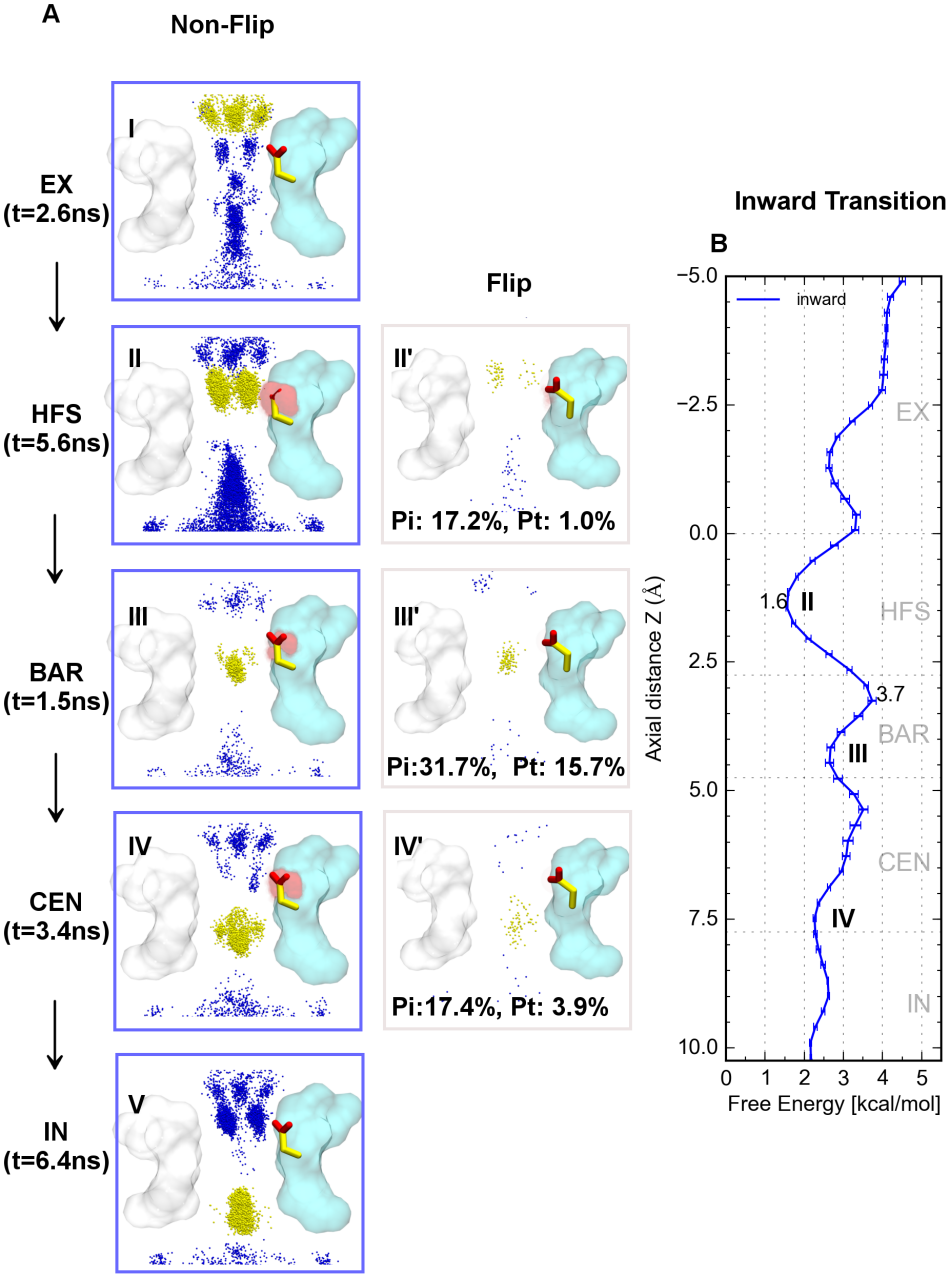
Inward ionic binding modes and PMF. A) Overlay of the conformational space of the probe ions (yellow), coupling sodium ions (blue) and E53 carboxyl oxygen distributions (transparent spheres with different intensity) for different ionic binding modes at different interaction sites. A snapshot of a typical E53 sidechain conformation is shown as stick representation (red, carboxyl oxygens; yellow, sidechain carbons), I–V are non-flip binding modes and II′–IV′ are flipping binding modes for sites S_HFS_, S_BAR_ and S_CEN_. Arrows indicate the direction of ion conduction; B) 1-D PMF of inward conduction in SF region, the largest free energy barrier is labeled by a terminal peak and well, corresponding positions of typical binding modes on the energy profile are labeled. Error estimations shown in the figure are S.E.M.

**Figure 7:**
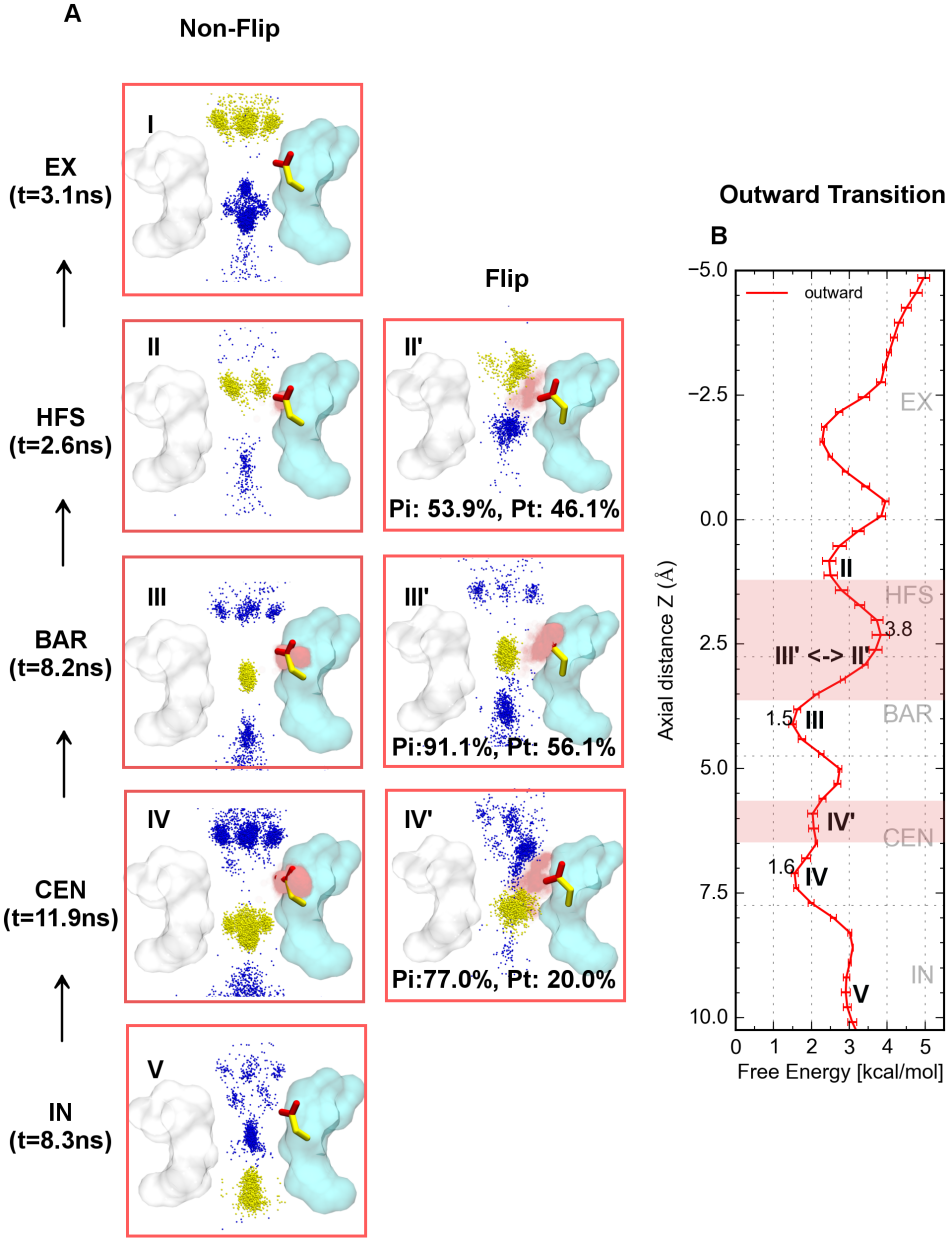
Outward ionic binding modes and PMF. A) Overlay of the conformational space of the probe ions (yellow), coupling sodium ions (blue) and E53 carboxyl oxygen distributions (transparent spheres with different intensity) for different ionic binding modes at different interaction sites. A snapshot of a typical E53 sidechain conformation is shown as stick representation (red, carboxyl oxygens; yellow, sidechain carbons), I–V are non-flip binding modes and II′–IV′ are flipping binding modes for sites S_HFS_, S_BAR_ and S_CEN_. Arrows indicate the direction of ion conduction; B) 1-D PMF of outward conduction in SF region, the largest free energy barriers are labeled by terminal peaks and wells, corresponding positions of typical binding modes on the energy profile are labeled. Error estimations shown in the figure are S.E.M.

A detailed investigation of the ionic binding modes and their relations to free energy profiles enabled us to describe mechanisms regarding different conducting directionalities (Figure 6 and 7). During inward conduction, the largest barrier in the SF occurs between sites S_HFS_ and S_BAR_ which amounts to 2.1 kcal/mol (Figure 6B). At site S_HFS_, the probe ions (yellow) mainly distributed in an off-axis manner, the first coupling Na^+^ ions (blue) may occupy site S_CEN_ _(IN)_, and there existed a second binding site for coupling ions at site S_EX_ (Figure 6A, II). Subsequently, the probe ions distributed in the middle of channel axis when traversing the short lived site S_BAR_, with the other two coupling ions populating sites S_EX_ and S_IN (CAV)_ respectively (Figure 6A, III). The probe ions then occupied site S_CEN_ in both on-axis and off-axis manners, other coupling ions in the SF were distributed mainly at sites S_IN_ and S_EX_. Only a few coupled ions occupied sites S_HFS_ and S_BAR_ (Figure 6A, IV).

In these ionic binding modes, under hyperpolarized potential, the coupling ions in the SF generally demonstrated a loosely coupled knock-on mechanism with only a few of them present in the adjacent binding sites to the probe ions (Figure 6A, II–IV). This is in agreement with a study by [21], [22], where it was shown that during inward conduction the ions displayed a combination of mono-ionic and multi-ionic mechanism with an overall occupancy of 1.8 ions in the pore region.

The flipping probability analysis indicates that the conformational changes of the E53 side chains play a minor role for ion inward conduction as shown in Figure 6A, II′–IV′.

### Ionic binding modes and conduction mechanism during outward conduction

Compared to inward permeation, the maximum energy barrier during outward conduction amounted to 2.3 kcal/mol between sites S_BAR_ and S_HFS_. It is interesting that the free energy difference between sites S_CEN_ and S_HFS_ is 2.2 kcal/mol, which is close to the largest energy barrier (Figure 7B). In addition, the ionic binding modes demonstrate a distinct conduction mechanism compared to Na^+^ influx. Traversing outward from the cavity, ions at site S_IN_ were tightly coupled with ions at site S_BAR_ (Figure 7A, III and V) corresponding to two energy wells in Figure 7B, III and V). When probe ions located at site S_CEN_, the coupling ions distributed in the upper part of the cavity (Figure 7A, IV) which corresponds to the energy well at site S_CEN_ (Figure 7B, IV). Generally, the translocation of probe ions from the cytoplasm to site S_BAR_ is readily stepwise by a tight knock-off mechanism without significant energy barriers. At all three energy wells (Figure 7B III–V) the E53 side chains maintained non-flipped conformations.

When probe ions faced the energy barrier at site S_BAR_, a delicate tightly-coupled “knock-off” conducting mechanism occurred. Initially, E53 started to flip and one of the carboxyl oxygens started to coordinate the probe ions (Figure 7A, III′ and S5, B). Compared to ions located in the close energy wells (Figure 7A, III), the probe ions were meanwhile expulsed by the outward movements of approaching coupling ions at site S_IN_ (Figure 7A, III′). If this knock-off mechanism was successful, the probe ions would then migrate to site S_HFS_, as a result, the coupling ions would move outward to site S_CEN_ simultaneous (Figure 7A, II′ and IV′). At this time, two carboxyl oxygens of the flipped E53 side chain tended to coordinate with the probe ions and coupling ions respectively (Figure 7A, II′ and IV′ and S5, B). Afterwards, ions left site S_HFS_ promptly (t  =  2.6 ns) into the periplasm via site S_EX_.

If the attempt to overcome the barrier failed, the aforementioned mechanism was easily reversed, the probe ions and coupling ions occupied the two stable energy wells at sites S_BAR_ with the coupling ions at site S_IN_ (Figure 7A, III and V) and site S_CEN_ with the coupling ions in the cavity (Figure 7A, IV) again. That is the reason why ions stayed longer in sites S_BAR_ and S_CEN_.

One the one hand, larger Pi values (flip inducing probability of number of probe ions, see methods for details) values of sites S_BAR_, S_CEN_ and S_HFS_ indicated the flipped conformations of E53 were crucial (Pi>90%) in overcoming the dual energy barriers between S_CEN_, S_BAR_ and S_HFS_. On the other hand, smaller Pt values (flipping time probability for all probe ions, see methods for details) values indicated that the flipping events were easily reversible. Because of these flipping events, the major ion distribution for outward conduction is limited to the center of the channel axis during translocation within the SF.

### E53 conformation determines efflux rate

**Figure 8:**
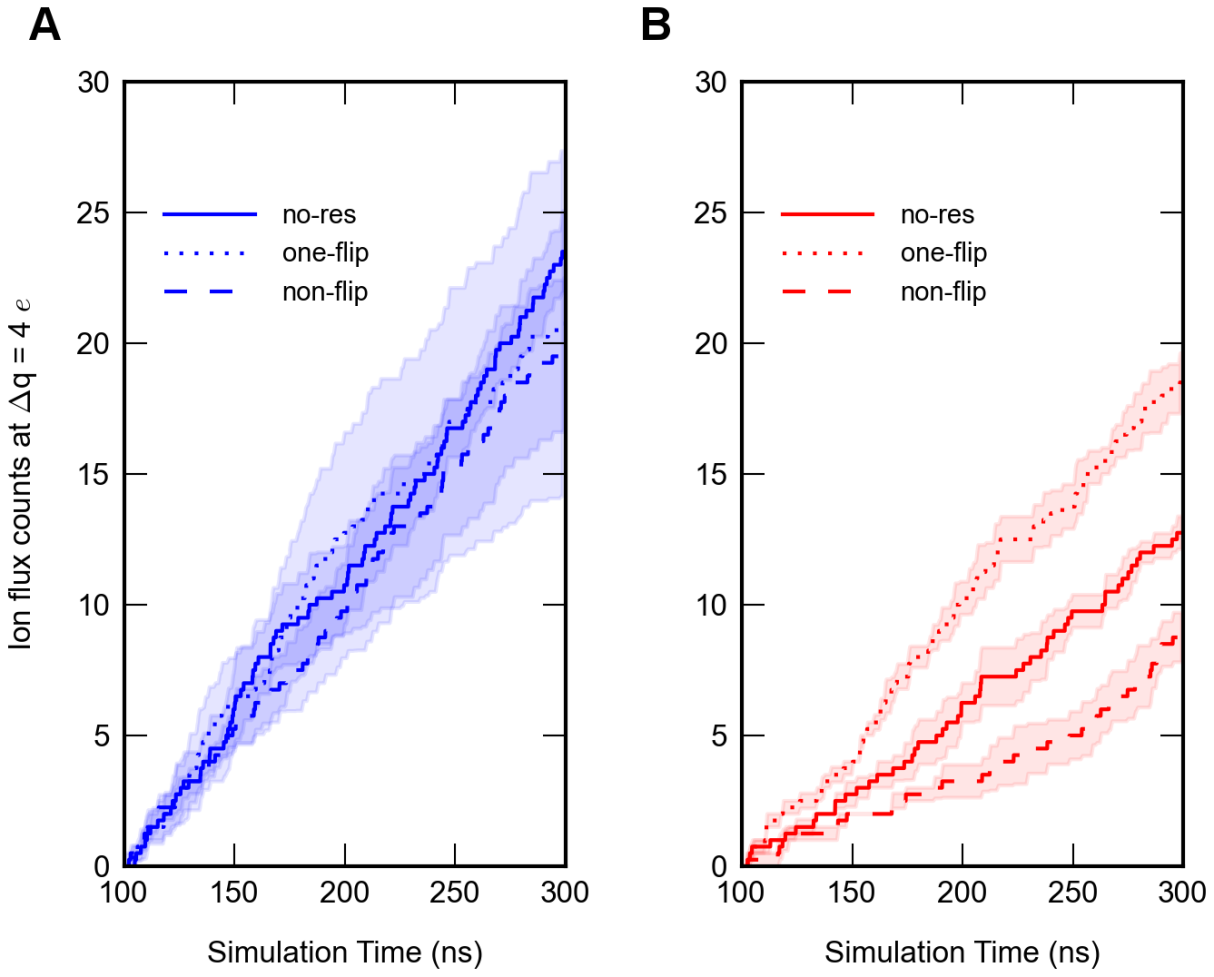
Influences of different flipping states at Δq =  4*e* (n = 4). A) Average ion flux counts through the SF over time of inward conduction (color: blue). The simulations without restraints of E53 are depicted as solid line, the ones with the “one-flip” restraints are shown as dotted line and the ones with the “non-flip” restraints are shown as dashed line. B) Average ion flux count through the SF over time of outward conduction (color: red). Error estimations shown in the figure are S.E.M.

To further explore the correlation between flux directionality and E53 conformation, we performed two sets of inward and outward conduction simulations (four times 300 ns) with dihedral restraints to maintain “non-flip” and “one-flip” configurations during sampling. The influx rate was independent of the E53 conformations as shown in Figure 8A. This observation disagrees with recent data from Chakrabarti et al. [23] on the Na_V_Ab channel. The outward conduction with “one-flip” simulation displayed an increased efflux rate compared to simulations without dihedral restraints on E53, where the flipping events would be reversible when conducting ions (Figures S1, S2, S3, S4). Interestingly, if E53 was restrained to a “non-flip” configuration, sodium ions translocation slowed down (Figure 8B). These results suggest a clear influence of filter dynamics on the efflux rate.

**Figure 9:**
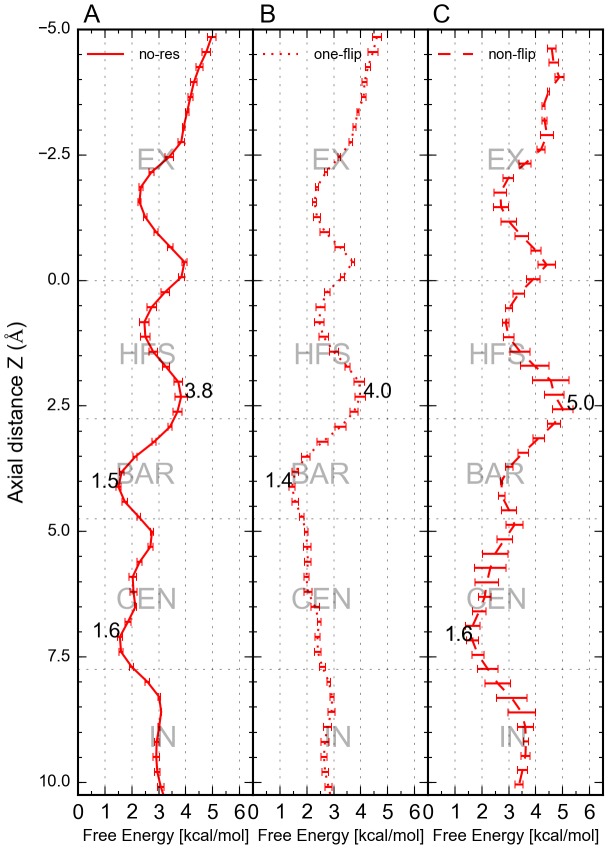
PMF comparison for outward conduction. A) PMF of the simulations without restraints of E53 is depicted as solid line. (B) PMF of the simulations with “one-flip” restraints. C) PMF of the simulations with “non-flip” restraints. The largest free energy barriers in each figure are labeled by terminal peaks and wells. Error estimations shown in the figure are S.E.M.

Comparison of the free energy profiles from outward simulations of these three types of configurations revealed that the largest energy barrier of the “non-flip” simulations is increased from 2.3 kcal/mol (non-restraint) to 3.4 kcal/mol (Figure 9A and C). The lowest energy well at site S_BAR_ was also replaced by site S_CEN_. The energy profile of “one-flip” simulations indicated that the energy barrier between sites S_CEN_ and S_HFS_ was diminished, although the original energy barrier increased slightly by 0.3 kcal/mol (Figure 9A and B). Therefore, the outward conduction would be more straightforward without reversible backward translocation compared to the simulations without the dihedral restraints from a kinetic point of view.

## Discussion

The mechanism of ion conduction and selectivity of bacterial voltage gated sodium channels is gradually emerging. The inverted tepee shape architecture of the SF lined with TLESW sequence enables sodium influx at the diffusion rate. The glutamic acid side chains are responsible for recruiting Na^+^ ions from the outer vestibule. Ions will then translocate via sites S_HFS_, S_BAR_, S_CEN_ and S_IN_ spontaneously to complete a conduction event [21]–[23]. Details of conduction are only partially understood. Large conformational changes of the glutamic acid side chains were described recently [23]. However, its role for conduction is still under discussion [24].

To gain further insights into these questions, we performed MD simulations to compare the different binding patterns and characterize the structural dynamics of glutamic acids during ion permeation. Double bilayer simulations with the open Na_V_Ms structure enabled us to investigate influx and efflux separately. The calculated inward conductance rate is in good agreement with a previously reported experiment and computational data [22]. The estimated outward conductance rate obtained from MD simulations is predicted to be markedly lower than inward permeation (15±3 pS vs 27±3 pS, Figure 1). Ion translocation between sites S_BAR_ and S_HFS_ is substantially prolonged (8.2±0.9 ns vs 1.5±0.3 ns, Figure 4) during Na^+^ efflux. From the energetic point of view, this would imply a potential barrier. This agrees with previous two-ion free energy calculation studies, revealing a higher energy barrier in this region for outward current compared to inward conduction (ΔG: 4.6 kcal/mol vs 0.4 kcal/mol, Stock et al. [21]; ΔG: 3.5±0.5 kcal/mol and 2.4±0.3 kcal/mol, Furini and Domene [15]). In our studies, this barrier is also higher for outward conduction (ΔG: 2.3 kcal/mol vs. 2.1 kcal/mol).

In agreement with previous studies [21], [22] during inward conduction, our simulations revealed that ion translocations in the SF generally involve a loosely coupled knock on mechanism with an average ion occupancy of 1.8 (Figure 6).

A possible outward conduction mechanism was described by Stock et al., [21] using a “fully activated-open” Na_V_Ab channel structure [28]. They have found a third ion denoted k, directly coupling with the probe ions triggering outward conduction by a “nudging” collision effect. Similar results were obtained in our study, which shows that the coupling ions directly couple with the probe ions by a tight “knock-off” mechanism. Moreover, our simulations further elucidated that this “knock-off” mechanism is highly dependent on the conformational isomerization of the glutamic acid side chains in the SF. In other words, to overcome the energy barriers of outward conduction, at least one of the glutamic acid side chains has to be flipped to an inward facing conformation (Figure 7).

A recent simulation study under ∼0 mV membrane potential with a closed gate Na_V_Ab structure suggested that Na^+^ in- and outward movement involves variable configurations of multiple glutamic acid side chains giving rise to non-simple degenerated ion binding modes [23]. Remarkably, detailed investigations of the structural dynamics of E53 in our study revealed distinct isomerization patterns between forward and backward translocations respectively. When the ion moved into the SF from the outer vestibule under hyperpolarized membrane potential, the E53 remained mostly in the non-flipped conformation. In contrast, during outward conduction, the flipping occurrence increased significantly with a typical “one-flip” configuration (Figure 5C and D) when coordinating ions occupied the SF (Figure 7). In our simulations, the depolarized and hyperpolarized membrane potentials of approximately ΔV: 565 mV enabled the detailed investigation of ion permeation directionalities. This was not possible in previous simulations at ∼0 mV [23]. The conductive, open gate structure used in this study may also reduce the repulsive effect which could have been induced by ions present in the cavity in previous simulations with a closed gate [23]. In addition, different forcefields used in these two studies may also play a substantial role for these discrepancies. As reported by Cordomi et al [29]), compared to the combination of OPLS-AA protein with Berger lipids parameters, combining Amber99sb protein and Berger lipids gives more accurate free energies of solvation in water and water to cyclohexane transfer with respect to experimental data for glutamic acid side chains. This may explain the reduced flexibility of the glutamic acid side chain dynamics observed in our study. Further, the force field discrepancies might explain the contrasting results for the “no salt” simulations in these two studies. In the study by Chacrabarti et al [23], the E side chains are more favorable to form flipped conformations even in the “no salt” conformation. This is in contrast to our simulations, where flipping events occurred rarely (Figure 5C) in the “no salt” simulations.

While the inward flow exhibited indistinguishable flux rates irrespective of the E53 conformation (Figure 8A), efflux displayed different rates depending on the configurations of the E53 side chain. The highest efflux rate was observed in our “one-flip” simulations and the lowest rate with all four glutamic acid side chains restrained to an outward-facing conformation (Figure 8B). PMF calculations further confirmed that the energy barrier for outward conduction increased from 2.3 kcal/mol to 3.4 kcal/mol when the flipping conformation is prohibited (Figure 9C). That indicates that this flipping conformation provides direct coordination for Na^+^ ions, which lowers the energy barrier and aids outward conduction.

A simulation study published [30] after the submission of this manuscript, indicates that the SF dynamics, especially the side chain conformational changes of the EEEE locus in the SF, may lead to the conformational changes of the cavity lining helix on the µs timescale, subsequently initiating slow inactivation in Na_V_ channels. We hypothesize that the E53 dynamics under depolarizing potentials uncovered in this study provide further insights into slow inactivation, especially the fast slow inactivation for prokaryotic species during action potentials. When the membrane potential depolarizes, the probability of Na^+^ outward transitions increases. As a result, the inactivation probability of the channel is increased probably due to a series of conformational changes starting from the EEEE locus in the SF.

A general limitation of current force fields is that the simulated linear current–voltage regime can only be achieved at higher membrane potentials compared to experimental conditions, resulting from the large electrostatic barriers in the transmembrane region [31], [32]. It should be noted that the computational electrophysiology simulations in this study were not done at constant membrane potentials (565±126 mV). This may result from the movement of the ions inside the channels and the fluctuation of the ions in the aqueous compartments (Figure 2A and B). However, a single ion permeation event under physiological conditions will also exist as a non-equilibrium process. Thus, to which extent, current simulation methods resemble ion channels' electrophysiology needs to be further validated. In addition, inaccuracies in the interaction parameters (from the forcefield) between ions and surrounding atoms could also influence the conduction rates [33]. Thus, further structural and computational studies (including optimized strategies for ion interaction with surrounding atoms and polarizable force fields with different lipid species) will be required to further investigate the conformational changes of the SF under different electrochemical drives and the influence of different protonation states of the EEEE locus. In addition, experimental validation is essential to further uncover the structural determinants and the importance of the protonation states of the EEEE locus on ion conductance and selectivity.

Summarizing, our simulations, using applied membrane potentials, reveal different conduction mechanisms for ion inward and outward transitions respectively. An inward facing conformation (flip) of one glutamic acid side chain in the SF would reduce the energy barrier for ion outward transition by providing direct coordination with interacting Na^+^ ions. This local change can provide insights into the slow inactivation of Na_V_ channels as suggested by Boiteux et al [30] during an action potential, when the membrane potential is depolarized.

## Methods

### MD simulations

The coordinates of Na_V_Ms (PDB Entry: 4F4L; Resolution: 3.49 Å) [7] with a conductive pore gate were used. The symmetric tetrameric structure consists of residues 8 to 94. All charged residues were treated keeping their charge states at physiological pH 7.4. In order to investigate ion conductance under two opposite membrane potentials, we used the computational electrophysiology method developed by Kutzner et al. [27] with a double-bilayer scheme. Each bilayer leaflet consists of 242 1-palmitoyl-2-oleoyl-sn-glycero-3-phosphocholine (POPC) lipids encompassing the protein structure, solvated with 500 mM NaCl solution. The system was then duplicated in the Z direction (pore axis). The virtual-site model was adopted for hydrogen atoms [34].

MD simulations were performed with Gromacs version 4.5.5-dev [35], [36]. Simulations were carried out with the AMBER99sb [37] all atom force field, POPC lipids parameter were taken from Berger et al. [29], [38] with the TIP3P water model [39]. All covalent bonds were constrained using the LINCS algorithm [40], allowing for an integration time step of 4 fs with virtual sites. A 10 Å cutoff was adopted for calculating short-range electrostatic interactions and the Particle Mesh Ewald [41] summation was used for calculating long-range electrostatic interactions. The corrected monovalent ion Lennard-Jones parameters for the amber forcefield [42] were implemented in this study and the vdW interactions were calculated with a cutoff of 10 Å. The Nose-Hoover thermostat [43], [44] and the semi-isotropic Parrinello-Rahman barostat algorithm [45] was used to maintain simulation temperature and pressure constantly at 300 K and 1 bar, respectively.

Prior to MD simulations, 3000 conjugate gradient energy-minimization steps were performed, followed by 5 ns equilibration in order to fully solvate mobile water and lipids around the restrained protein with a force constant of 1000 kJ/mol/nm^2^ on all heavy atoms. Hereafter, an equal number of Na^+^ ions and a net difference of 4 *e* of Cl^−^ across each lipid bilayer between the central electrolyte bath and the two outer ones were sustained during the simulation by a swapping mechanism [27]. In this scheme, a new form of Poisson equation [46] was adopted to derive the potential profile as a function of system length (z). The well-defined transmembrane voltage across each lipid patch was directly assessed by twice integration of this sustained charge density differences between the central electrolyte bath and the two outer ones [27]. In our simulations, depolarized and hyperpolarized membrane potentials were calculated as ΔV  =  565±126 mV (Figure 2). Harmonic restraints (1 kcal/mol/Å^2^) were exerted on the α-carbon atoms of the TM helices (S5 and S6) throughout the simulations to maintain the open configuration in the absence of the voltage-sensing domain as suggested by Ulmschneider et al. [22]. Four times 500 ns MD simulations were performed; the first 100 ns were treated as equilibration. Simulation trajectories were saved every 100 ps; as a result, 4000 snapshots (analyzing windows) were recorded for analyzing data. Three repeated 500 ns simulations (400 ns were adopted for analysis) with only ions neutralizing the system and no ions in the SF (“no salt”) were performed as control to investigate the influence of the membrane potential on the E53 side chain dynamics. In this setup, only ions used to generate the charge imbalance and neutralize the net charge were kept. Further, four repeated 300 ns simulation (200 ns were adopted for analysis) for “non-flip” and “one-flip” configurations respectively were carried out, where the dihedral restraints were applied on the χ_2_ dihedral of E53 for all four subunits with a force constant of 500 kcal/mol/rad^2^. This allows dynamic ranges of 56±10° for “one-flip” configuration and 288±10° for “non-flip” configuration of the χ_2_ angle.

### Ionic binding modes

The total number of ions (i) which completed their conduction in the SF were analyzed (i  =  158, from four inward simulations; and i  =  79 from four outward simulations). All snapshots of the probe ions (yellow) and coupling ions in the SF (blue) were rendered for five different interaction sites (S_EX_, S_HFS_, S_BAR_, S_CEN_ and S_IN_). For sites S_HFS_, S_BAR_ and S_CEN_, the side chain isomerization states were separated into flipped and non-flipped categories and analyzed. For flipped ones, all four protein chains and the relative ion positions were aligned to chain A to achieve a better representation of the ionic binding patterns. Two probability parameters Pi and Pt were calculated to characterize the influence of E53 dynamics on ion conduction for these three sites. Pi  =  (Fi/i)*100%, where Fi denotes the number of ions which generated at least one E53 flipping event during their permeation through each site. Pt =  Ft/Tt*100%, where Ft denotes the number of snapshots where E53 flipped when the probe ions traversed each site and Tt denotes the total number of snapshots when the probe ions traversed each site.

### Potential of mean force (PMF)

The 1-D potential of mean force profile of the ions under membrane potentials were calculated by taking the logarithm of the Na^+^ probability distribution along the channel axis (z) in the SF region, according to G(z)  =  −*k*_B_ *T* ln [*p*(*R_i_*)], where *k*_B_ is the Boltzmann constant, T is the temperature, and *p*(*R_i_*) is the probability distribution of the probe ions. 100 bins were used to achieve a bin width of 0.15 Å depicting the details of the profile. Error bars are S.E.M. from four different simulations.

## Supporting Information

Figure S1 **Cumulative ion flux counts with flip counts as a function of time from simulation 1 without dihedral restrains of E53.** A) & C) Ion flux counts through the SF and flipping counts of E53 over 400 ns trajectory of inward simulation (color: blue). B) & D) Ion flux counts through the SF and flipping counts of E53 in over 400 ns trajectory of outward simulation (color: red).
(TIFF)

Figure S2 **Cumulative ion flux counts with flip counts as a function of time from simulation 2 without dihedral restrains of E53.** A) & C) Ion flux counts through the SF and flipping counts of E53 over 400 ns trajectory of inward simulation (color: blue). B) & D) Ion flux counts through the SF and flipping counts of E53 in over 400 ns trajectory of outward simulation (color: red).
(TIFF)

Figure S3 **Cumulative ion flux counts with flip counts as a function of time from simulation 3 without dihedral restrains of E53.** A) & C) Ion flux counts through the SF and flipping counts of E53 over 400 ns trajectory of inward simulation (color: blue). B) & D) Ion flux counts through the SF and flipping counts of E53 in over 400 ns trajectory of outward simulation (color: red).
(TIFF)

Figure S4 **Cumulative ion flux counts with flip counts as a function of time from simulation 4 without dihedral restrains of E53.** A) & C) Ion flux counts through the SF and flipping counts of E53 over 400 ns trajectory of inward simulation (color: blue). B) & D) Ion flux counts through the SF and flipping counts of E53 in over 400 ns trajectory of outward simulation (color: red).
(TIFF)

Figure S5 **Hydration shell of the simulations without dihedral restrains of E53.** (A) Oxygen coordination numbers in the first hydration shell for inward sodium conduction (Oxygen atoms closer than 3.0 Å to Na^+^ were considered coordinating atoms): the total coordination number is depicted in grey; water oxygens are depicted as blue lines. Protein backbone oxygens are shown in green. E53 side chain oxygens are colored red. B) Coordination oxygen atoms numbers for outward sodium conduction.
(TIFF)

Movie S1 **The movie shows the double bilayer simulation scheme (400 ns).**
(MP4)

Movie S2 **A “flipped” trajectory clip showing one example conduction event from an outward simulation.** Two opposite subunits are shown as cyan sticks for clarity. The E53 side chains are highlighted in yellow. Sodium ions are shown in yellow, except for Na^+^ ions in the SF, which are colored in different colors. The water molecules within 3 Å of these ions are represented as sticks to depict the hydration shell.
(MP4)
