# Supplementary figures and images for: Different Inward and Outward Conduction Mechanisms in NaVMs Suggested by Molecular Dynamics Simulations
Source: PLoS Comput Biol. 2014 Jul 31;10(7):e1003746. doi: 10.1371/journal.pcbi.1003746 (PMC4117422; doi:10.1371/journal.pcbi.1003746)

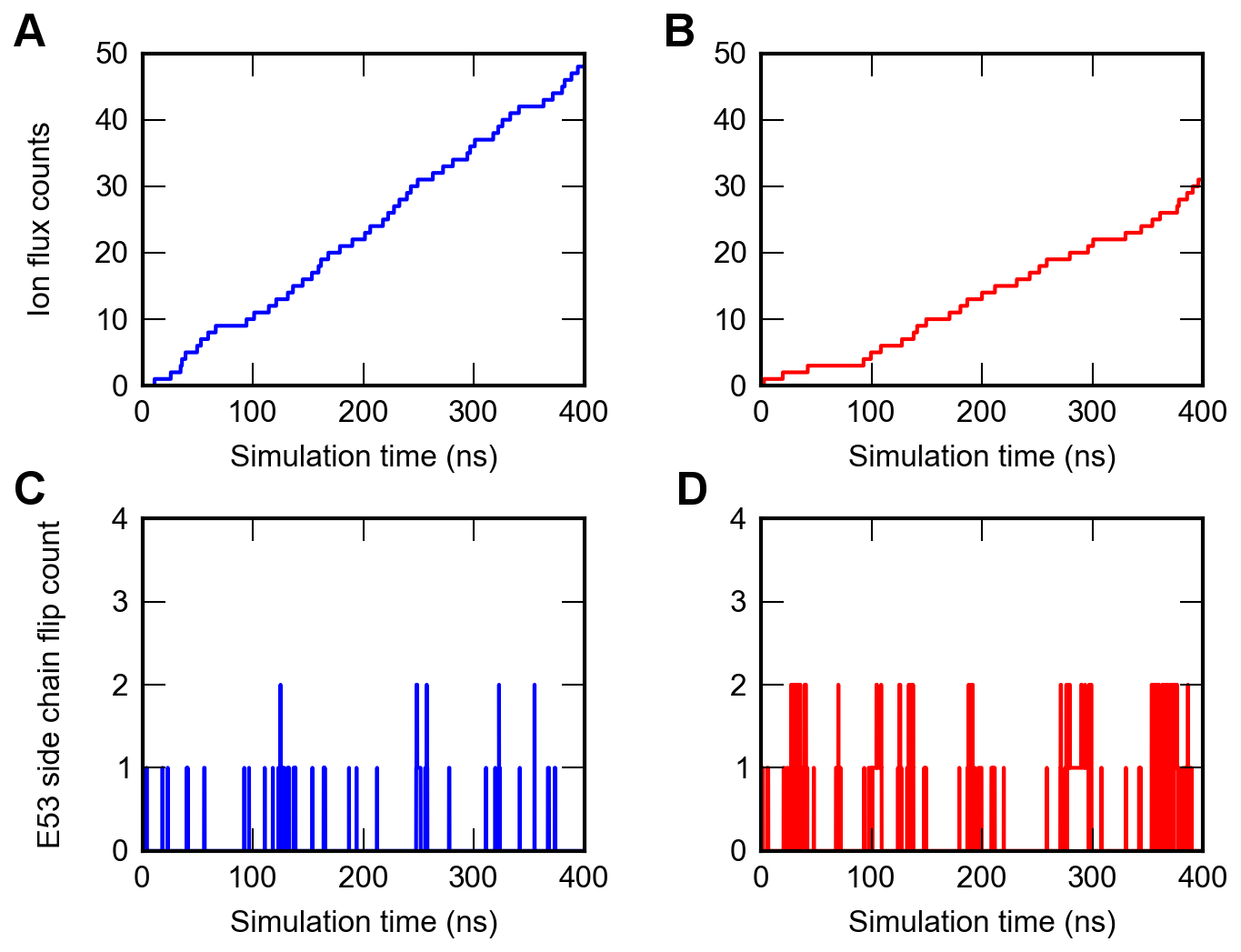

Supplement: Figure S1 — Cumulative ion flux counts with flip counts as a function of time from simulation 1 without dihedral restrains of E53. A) & C) Ion flux counts through the SF and flipping counts of E53 over 400 ns trajectory of inward simulation (color: blue). B) & D) Ion flux counts through the SF and flipping counts of E53 in over 400 ns trajectory of outward simulation (color: red). (TIFF) [file pcbi.1003746.s001.tiff]

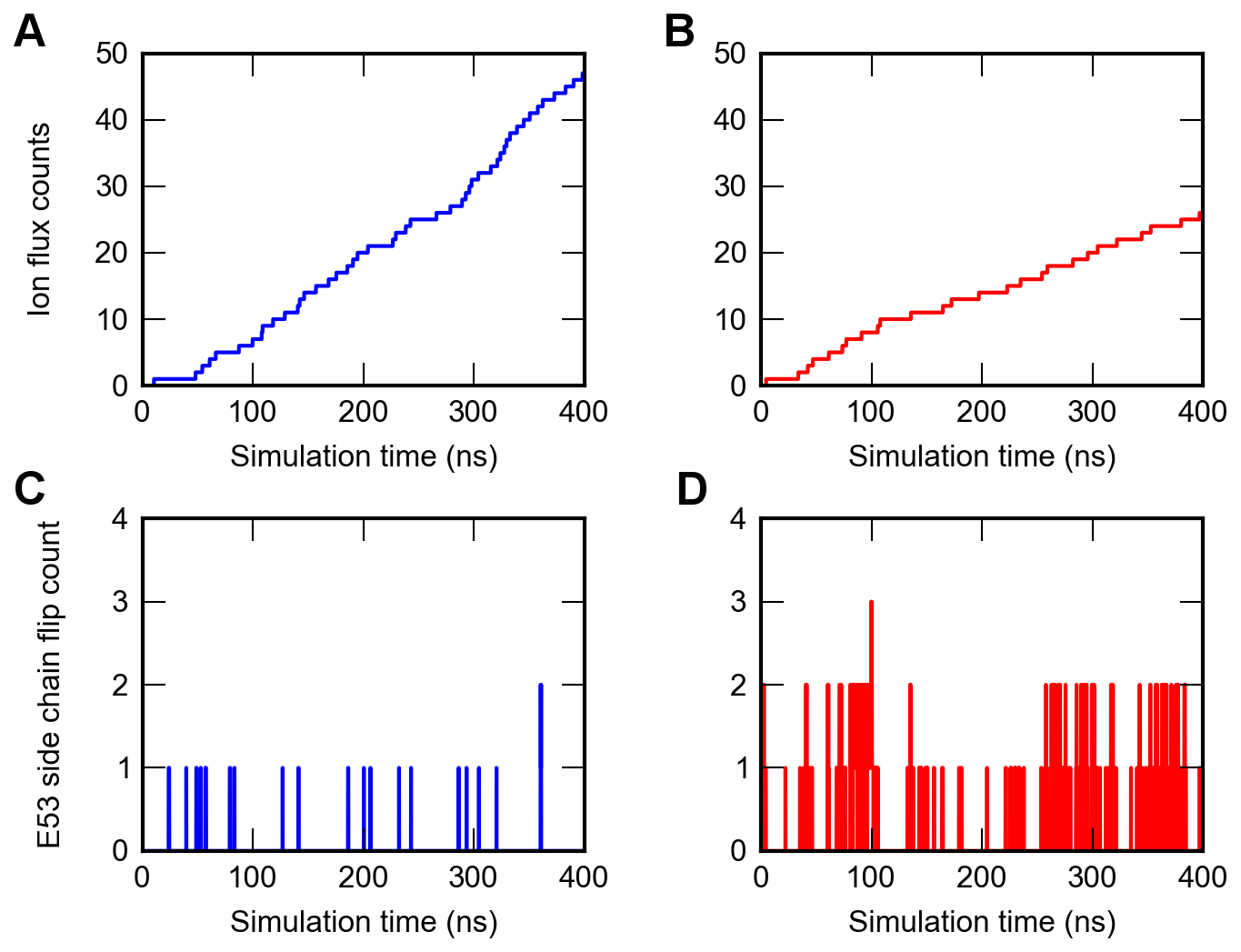

Supplement: Figure S2 — Cumulative ion flux counts with flip counts as a function of time from simulation 2 without dihedral restrains of E53. A) & C) Ion flux counts through the SF and flipping counts of E53 over 400 ns trajectory of inward simulation (color: blue). B) & D) Ion flux counts through the SF and flipping counts of E53 in over 400 ns trajectory of outward simulation (color: red). (TIFF) [file pcbi.1003746.s002.tiff]

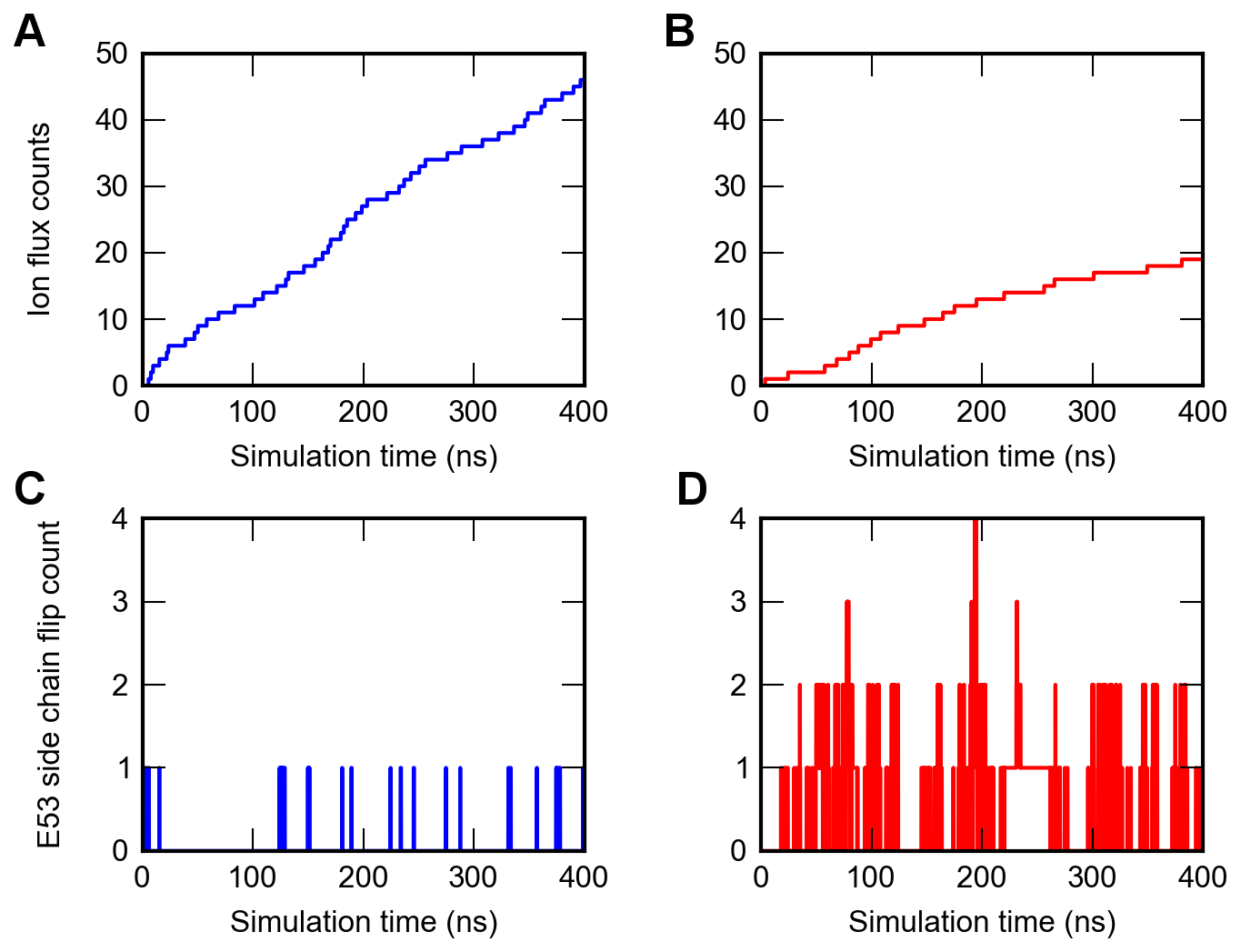

Supplement: Figure S3 — Cumulative ion flux counts with flip counts as a function of time from simulation 3 without dihedral restrains of E53. A) & C) Ion flux counts through the SF and flipping counts of E53 over 400 ns trajectory of inward simulation (color: blue). B) & D) Ion flux counts through the SF and flipping counts of E53 in over 400 ns trajectory of outward simulation (color: red). (TIFF) [file pcbi.1003746.s003.tiff]

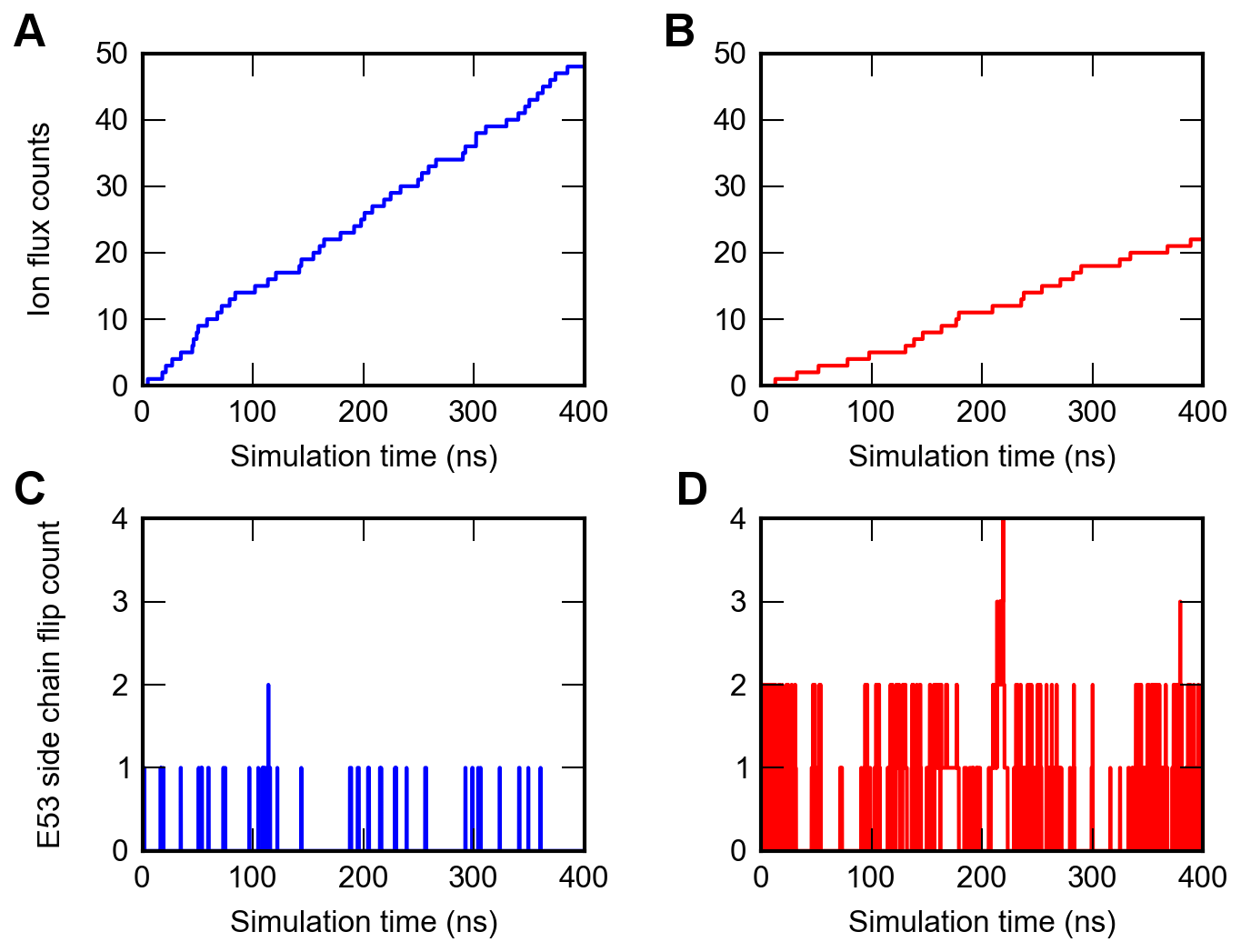

Supplement: Figure S4 — Cumulative ion flux counts with flip counts as a function of time from simulation 4 without dihedral restrains of E53. A) & C) Ion flux counts through the SF and flipping counts of E53 over 400 ns trajectory of inward simulation (color: blue). B) & D) Ion flux counts through the SF and flipping counts of E53 in over 400 ns trajectory of outward simulation (color: red). (TIFF) [file pcbi.1003746.s004.tiff]

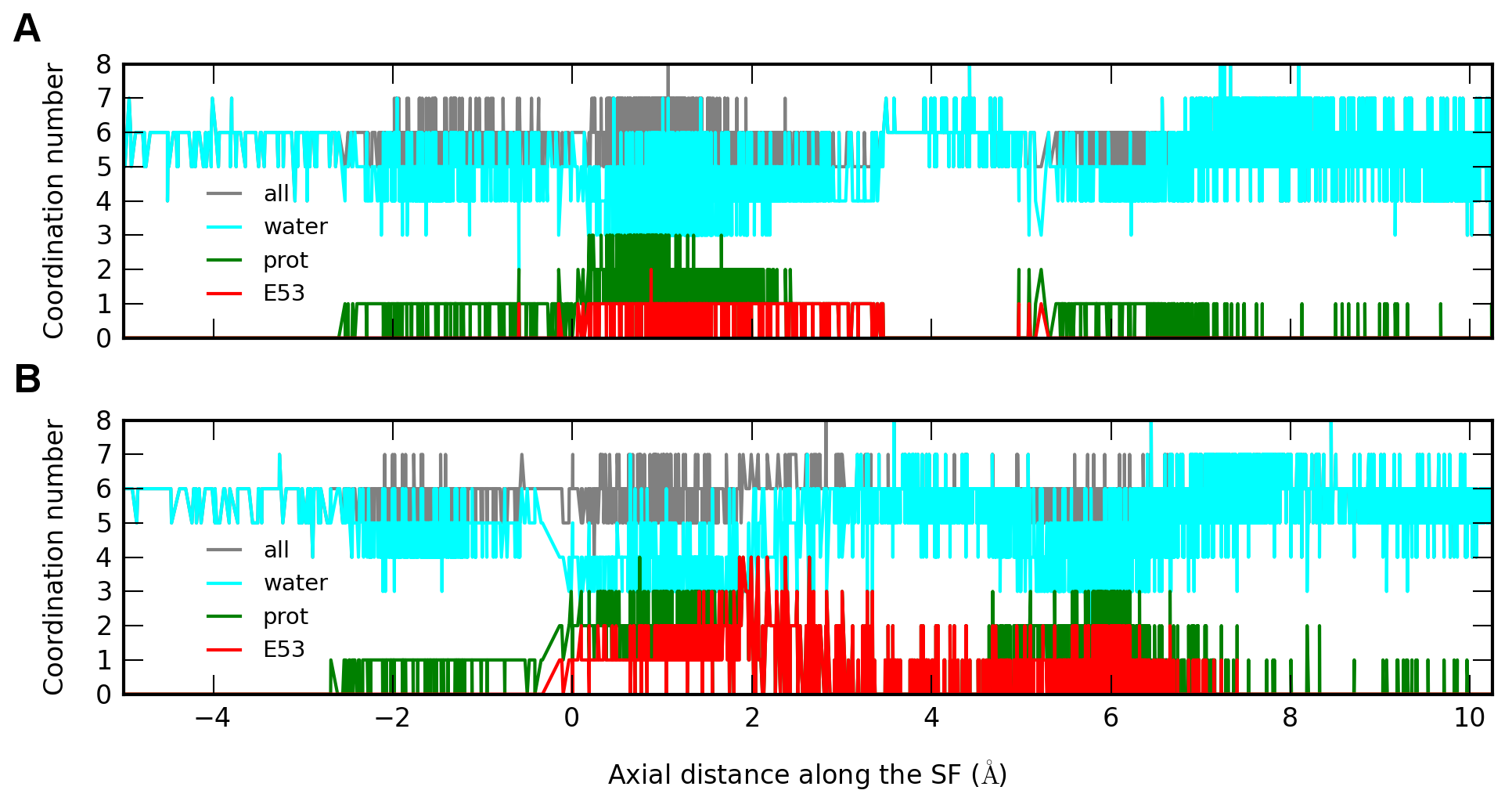

Supplement: Figure S5 — Hydration shell of the simulations without dihedral restrains of E53. (A) Oxygen coordination numbers in the first hydration shell for inward sodium conduction (Oxygen atoms closer than 3.0 Å to Na+ were considered coordinating atoms): the total coordination number is depicted in grey; water oxygens are depicted as blue lines. Protein backbone oxygens are shown in green. E53 side chain oxygens are colored red. B) Coordination oxygen atoms numbers for outward sodium conduction. (TIFF) [file pcbi.1003746.s005.tiff]
